# Robust Low-Snapshot DOA Estimation for Sparse Arrays via a Hybrid Convolutional Graph Neural Network

**DOI:** 10.3390/s25154563

**Published:** 2025-07-23

**Authors:** Hongliang Zhu, Hongxi Zhao, Chunshan Bao, Yiran Shi, Wenchao He

**Affiliations:** 1 College of Communications Engineering, Jilin University, Changchun 130015, China; zhuhl24@mails.jlu.edu.cn (H.Z.); hxzhao24@mails.jlu.edu.cn (H.Z.); baocs24@mails.jlu.edu.cn (C.B.); 2 School of Mechanical and Electrical Engineering, Changchun Humanities and Sciences College, Changchun 130117, China

**Keywords:** direction-of-arrival (DOA) estimation, graph neural network, low snapshots, array signal processing

## Abstract

We propose a hybrid Convolutional Graph Neural Network (C-GNN) for direction-of-arrival (DOA) estimation in sparse sensor arrays under low-snapshot conditions. The C-GNN architecture combines 1D convolutional layers for local spatial feature extraction with graph convolutional layers for global structural learning, effectively capturing both fine-grained and long-range array dependencies. Leveraging the difference coarray technique, the sparse array is transformed into a virtual uniform linear array (VULA) to enrich the spatial sampling; real-valued covariance matrices derived from the array measurements are used as the network’s input features. A final multi-layer perceptron (MLP) regression module then maps the learned representations to continuous DOA angle estimates. This approach capitalizes on the increased degrees of freedom offered by the virtual array while inherently incorporating the array’s geometric relationships via graph-based learning. The proposed C-GNN demonstrates robust performance in noisy, low-data scenarios, reliably estimating source angles even with very limited snapshots. By focusing on methodological innovation rather than bespoke architectural tuning, the framework shows promise for data-efficient DOA estimation in challenging practical conditions.

## 1. Introduction

Direction-of-Arrival (DOA) estimation is a key problem in signal processing of arrays, with widespread applications in radar [1,2], sonar [3,4], wireless communications [5,6], underwater acoustic detection [7,8], location of acoustic sources [9], and beamforming [10]. Accurate estimation of DOA not only aids in better signal separation and enhancement but also significantly improves the spatial resolution of the system. For example, DoA estimation provides precise target and interference directions for beamforming [11], allowing the array to steer its main lobe toward the desired signal and suppress interference with its side lobes, thereby markedly improving spatial focusing gain and sensing performance.Traditional DOA estimation algorithms, such as MUSIC [12], ESPRIT [13], orthogonal matching pursuit(OMP) [14], and the Capon beamformer [15], perform well with uniform linear arrays (ULAs) under a large number of snapshots.In practical systems, due to constraints on cost, space, and power, array sensors are often sparsely deployed, limiting the number of snapshots [16] that can be obtained. Furthermore, the high-speed motion of the detected targets [17] or anti-surveillance requirements may restrict signal collection to very brief periods, resulting in only a few snapshots being captured. These factors lead to poor estimation of the sample covariance matrix, causing a sharp degradation in the performance of conventional methods under such conditions.

To overcome the grid mismatch problem inherent in traditional compressive sensing-based DOA estimation, two representative gridless approaches have been proposed: sparse Bayesian learning (SBL) [18,19] and atomic norm minimization (ANM) [20]. SBL adopts a hierarchical probabilistic framework that adaptively learns sparsity-promoting priors, allowing for off-grid refinement through iterative inference. ANM, on the other hand, formulates DOA estimation as a convex optimization problem over the continuous domain, achieving super-resolution without requiring discretization. However, SBL typically involves computationally intensive matrix inversions [21] and suffers from scalability issues in large arrays [22], while ANM requires solving large-scale semidefinite programs [23], which limits its practicality in real-time scenarios.

To overcome the angular resolution bottleneck caused by a limited number of sensors, reference [24] proposes an all-digital synchronized AOA estimation architecture using phase interferometry, which enables high-precision real-time angle estimation with a small number of array elements. Sparse array techniques [25] design sensor locations to achieve degrees of freedom and resolution higher than those of uniform linear arrays. In particular, difference coarray theory [26] provides a means to map spatial difference information from a sparse array to VULA, and its effective array aperture is larger than that of the physical array, enabling the use of fewer physical sensors and thereby reducing design costs. At the same time, the increased degrees of freedom make it possible to perform multi-source DOA estimation in undersampled settings where the number of sources exceeds the number of array elements. This benefit, however, relies on arranging the elements according to an appropriate pattern; otherwise, holes will appear in the virtual array. Moreover, under low-snapshot conditions, the covariance matrix formed from a sparse array is often rank-deficient, making it difficult for subspace methods to reliably extract signal features, resulting in a marked drop in estimation accuracy.

Recent advances in deep learning have shown remarkable promise for signal-processing tasks by training neural networks to learn nonlinear mapping from array outputs to the spatial coordinates of signal sources, thereby predicting their incident angles with high accuracy. These networks include DNNs [27], convolutional recurrent neural networks (CRNNs) [28] and graph neural networks (GNNs) [29]. In particular, GNNs [30] excel at modeling intricate node-to-node relationships and have proven highly effective in exploiting the structured information embedded in array covariance matrices. Unlike conventional convolutional neural networks (CNNs) [31], which assume grid-structured inputs, GNNs represent data through adjacency matrices and can therefore capture long-range dependencies in non-Euclidean domains. By integrating one-dimensional convolution layers [32], which distill fine-grained local spatial features, with GNN-based modules for global structural learning, we obtain a hybrid feature-extraction architecture that substantially improves DOA estimation accuracy for sparse arrays under low-snapshot conditions.

Moreover, conventional classification-based DOA estimation methods [33] quantize the angle domain, which unavoidably introduces quantization errors and involves considerable computational complexity. In contrast, a regression-based deep learning model offers a more fine-grained end-to-end solution: it can directly output continuous angle values, thereby improving estimation precision and better accommodating scenarios with multiple simultaneous sources.

The paper is organized as follows: Section 2 explains the physical principles underlying DOA signals and proposes the sparse-array signal model that constitutes the focus of this study. Using the difference–coarray technique, an VULA is synthesized, effectively enlarging the aperture and improving the DOA resolution. The chapter also introduces the operational concepts of GNN and details the implementation of one-dimensional convolutions for local feature extraction. Section 3 substantiates the proposed approach under conditions of low snapshot and low signal-to-noise ratio. Its efficacy is demonstrated through scatter plot visualizations, comparative experiments, and quantitative accuracy evaluations. We also conducted physical experiments in this section to validate the C-GNN model’s ability to process real signals. Section 4 summarizes the main contributions and findings of this work.

## 2. Materials and Methods

### 2.1. Signal Model

In this work, we consider *M* far-field, narrowband, uncorrelated source signals impinging on an array, as described by the following equation:

(1) $$\theta ={\left[\theta_{1},\theta_{2},\ldots ,\theta_{M}\right]}^{⊤}$$

For a sparse sensor array with *N* sensors, let the set of sensor positions be $L=\{l_{1},l_{2},\ldots ,l_{N}\}$. The signal received by the *n*-th sensor at time *t* can be expressed as follows [34]:

(2) $$\psi_{n}\left(t\right)=\sum_{k=1}^{M}a_{n}\left(\theta_{k}\right)s_{k}\left(t\right)+n_{y}\left(t\right)$$

where the contribution of each source and noise is accounted for. The complete array observation vector is written as follows:

(3) $$a_{n}\left(\theta_{k}\right)=e^{-j2\pi /\lambda \left(n-1\right)d\sin\left(\theta_{k}\right)}$$

In these expressions, the steering vector a(θ) represents the array response for a source at direction θ, and other symbols are defined accordingly. The overall observation vector is written as follows:

(4) $$\psi \left(t\right)=A\left(\theta \right)s\left(t\right)+n_{y}\left(t\right)$$

where

(5) $$A\left(\theta \right)=\left[a\left(\theta_{1}\right),a\left(\theta_{2}\right),\ldots ,a\left(\theta_{M}\right)\right]$$

To improve the DOA estimation accuracy of the sparse array, we employ the difference coarray method to construct an VULA. Specifically, we compute the distance between every pair of sensor positions in *L*:

(6) $$\Delta =\left\{|n_{i}-n_{u}|:n_{i},n_{u}\in \chi \right\}$$

After removing duplicates, we obtain an unique non-negative distance set *D*. The number of elements in *D* determines the number of elements in the virtual array. The positions of the virtual array elements are then given as follows:

(7) $$P^{\prime }=\left\{p_{1}^{\prime },p_{2}^{\prime },\ldots ,p_{\left|D\right|}^{\prime }\right\},$$

as derived from the difference set. In our experiments, we consider a particular sparse array geometry for

**Figure 1:**
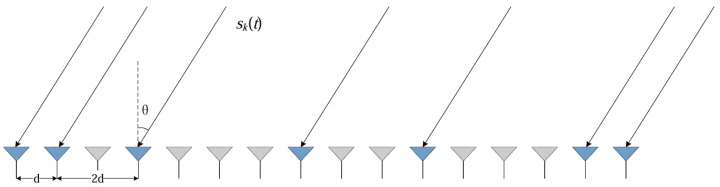
Schematic illustration of the impinging signals for a sparse array: red markers denote the actual antenna elements, whereas gray markers indicate the virtual elements generated via the difference-coarray technique.

(8) L={0,1,3,7,10,14,15},

As shown in Figure 1, This array configuration can construct a hole-free 16-element virtual uniform linear array using the minimum possible number of physical elements. The corresponding virtual array $P^{\prime }$ spans a larger effective aperture. For a single signal source with DOA θ, the sparse array’s steering vector is as follows:

(9) $$a\left(\theta_{k}\right)={\left[1,e^{-j2\pi /\lambda d\sin(\theta_{k})},e^{-j2\pi /\lambda 3d\sin(\theta_{k})},\ldots ,e^{-j2\pi /\lambda 15d\sin(\theta_{k})}\right]}^{T}$$

And its conjugate transpose is $a^{*}\left(\theta \right)$. Using the Kronecker product of the steering vector with its conjugate (i.e., $a\left(\theta \right)⊗a^{*}\left(\theta \right)$), we can construct the autocorrelation structure of the received signal. After removing duplicate entries corresponding to redundant or repeated difference lags, we obtain the virtual array steering vector $a^{\prime }\left(\theta \right)$ associated with the virtual array $P^{\prime }$. Consequently, we can determine the positions $P^{\prime }$ and the steering vectors for the VULA.

Using the virtual array, the covariance matrix of the sparse array observations can be expressed analytically. For example, a model for the covariance [35] is given as follows:

(10) $$R=A\;R_{s}\;A^{H}+\sigma_{n}^{2}I,$$

where *A* is the steering matrix in full array constructed from the steering vectors of all sources, $R_{s}$ is the covariance matrix of the source signal, $\sigma_{n}^{2}$ is the noise variance, and *I* is the identity matrix.

Because modern neural networks have difficulty processing complex-valued data directly, we convert the complex covariance matrix *R* into a real-valued representation. We do this by separating the real and imaginary parts of *R* and then concatenating them to form the final input matrix. In other words, we represent the input as a combination of the real-part matrix $R^{\left(r\right)}$ and the imaginary-part matrix $R^{\left(i\right)}$ of *R*.

### 2.2. C-GNN Model

Low-snapshot signal data contains very limited information, which leads to an estimated covariance matrix with insufficient rank. Traditional DOA methods rely on an accurate covariance matrix estimate and therefore suffer severe performance degradation in such cases. To effectively handle DOA estimation with few snapshots, we propose a GNN model with an embedded one-dimensional convolution, which processes the covariance matrix to extract both local and overall structural features.

In this design, the data of the matrix in the form of the input of the covariance matrix is represented as a graph G=(V,E), where *V* is the set of nodes containing feature vectors and *E* is the set of edges representing connectivity between nodes. Information can propagate between nodes along these edges. Typically, the connections are encoded by an adjacency matrix that describes which nodes are linked.

The adjacency matrix *B* in our model is an f×f matrix, where *f* is the number of nodes. It represents the connectivity structure of the graph *G*. The elements of *B* are defined follows:

(11) $$B=\left[B_{i,u}\right]\in {\left\{0,1\right\}}^{f\times f}$$

**Figure 2:**
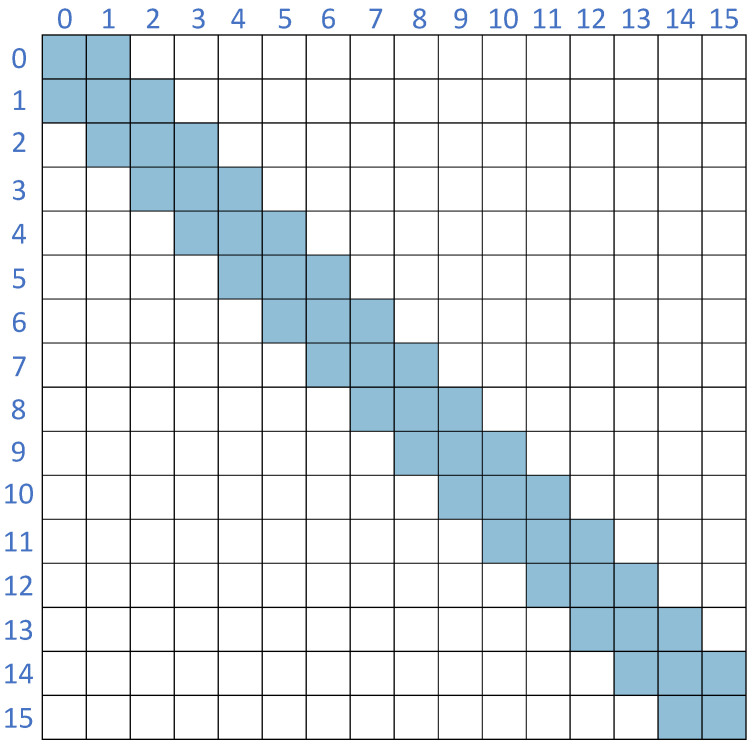
Schematic of the adjacency matrix, where blue means connected and white means unconnected.

Because we construct an undirected graph, for any pair of nodes $v_{i}$ and $v_{j}$, if an edge $\left(v_{i},v_{j}\right)$ exists, then an edge $\left(v_{j},v_{i}\right)$ also exists. Thus, the adjacency matrix is symmetric as shown in Figure 2.

In our graph formulation, we treat each row of the covariance matrix as a node feature vector. Therefore, the total number of nodes *f* is equal to the number of rows or columns of the covariance matrix. We then connect nodes corresponding to adjacent rows with edges, which facilitates information passing between neighboring rows. Each node is connected to its immediate neighbors in the matrix index, forming a chain-like graph structure.

The propagation of information between the connected nodes is performed along these edges.

(12) $$\Omega_{i}=\sum_{u\in N(i)}B_{i,u}\;h_{u}.$$

Here, the aggregated message for a node is calculated from the characteristics of its neighboring nodes weighted by adjacency connections.

We adopt a Graph Convolutional Network (GCN) as the specific GNN architecture in our model. The update rule for the *l*-th layer of the GCN [36] is given in Equation (13). In matrix form, a typical GCN layer operation can be written as follows:

(13) $$H^{\left(l\right)}=\sigma \;\left(B\;H^{\left(l-1\right)}W^{\left(l\right)}+b^{\left(l\right)}\right)$$

where $H^{\left(l\right)}$ is the matrix of node feature vectors at layer *l*, $H^{\left(l-1\right)}$ is the feature matrix from the previous layer, $W^{\left(l\right)}$ and $b^{\left(l\right)}$ are the weight matrix and bias of layer *l*, and σ(·) is a nonlinear activation function ReLU.

(14) $$\mathrm{ReLU}\left(x\right)=\max\left(0,\;x\right)=\left\{\begin{array}{cc} 0, & x<0,\\ x, & x\geq 0. \end{array}\right.$$

**Figure 3:**
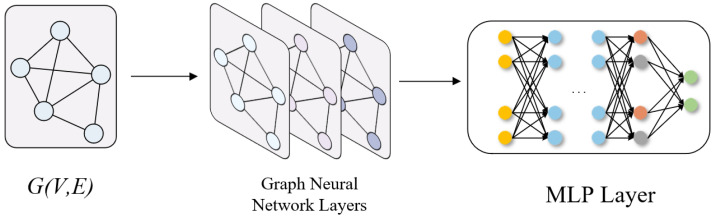
The process of information propagation and pooling process in the GNN.

This graph convolution aggregates information from each node’s neighbors (as encoded in B) and updates the node features. Figure 3 illustrates the process of information propagation and pooling process in the GNN.

After several graph convolution layers, we apply a global pooling operation to aggregate the node features into a single graph-level feature vector for final estimation. The pooling operation is defined in Equation (15). For example, a simple pooling strategy is to sum or average the feature vectors of all nodes:

(15) $$g=pooling\left(h_{global}\right)=\sum_{i=1}^{f}h_{i}^{\left(L\right)}$$

where *g* is the global pooling feature vector, $h_{i}^{\left(L\right)}$ is the feature vector of node *i* from the last GNN layer *L*, and *f* is the total number of nodes. This pooling vector g represents the aggregated information of the entire graph and will be used for the DOA regression.

Most DOA estimation algorithms typically require at least 400 snapshots to achieve accurate results [37,38]. Insufficient snapshots may lead to larger estimation errors in the covariance matrix, thereby introducing significant inaccuracies. Due to the limited number of snapshots, the information within each node may not be sufficiently distinctive on its own. To address this, we incorporate a one-dimensional convolution (1D convolution) as a preprocessing step before graph convolution. Specifically, we perform a 1D convolution along the dimension of the node of the input matrix. The goal is to extract local spatial features among neighboring virtual array elements, which complements the global structural features learned by the GNN layers. This design leverages the local receptive field ability of CNNs together with the GNN’s ability to model global relationships.

**Figure 4:**
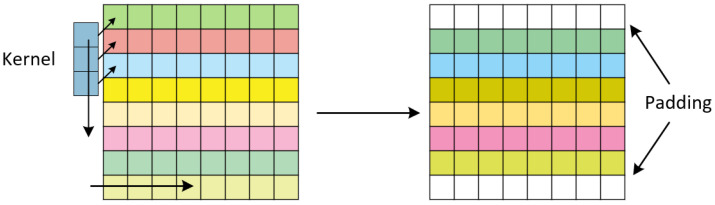
1D convolution process along the virtual array (node) dimension.

As shown in Figure 4, we use a convolution kernel of size 1 × 3 that slides along the row dimension of the matrix. We apply appropriate zero-padding at the boundaries to maintain the consistency of input and output matrix dimensions. This convolution produces an enhanced feature matrix which is then used as the node features input to the GNN. By combining this CNN-based local feature extractor with the GNN, we form the hybrid C-GNN model that can effectively capture both local and global patterns in the data.

### 2.3. MLP Regression Module

After the graph convolution layers and pooling layer, the feature representation obtained from the C-GNN is finally fed into an MLP (Multi-Layer Perceptron) to predict the DOA angles. Unlike many deep learning-based DOA approaches that perform classification on discretized angle bins, which introduces quantization error and higher complexity, our model uses a regression approach to output continuous-valued angle estimates directly. This avoids quantization errors and provides better compatibility with scenarios involving multiple simultaneous sources, as the network can output multiple angle values without needing a classification grid.

The MLP consists of *L* fully connected layers, where the first L−1 layers are hidden layers and the final layer is the output layer. Each hidden layer applies a linear transformation followed by layer normalization (LN) and a ReLU activation.

**Figure 5:**
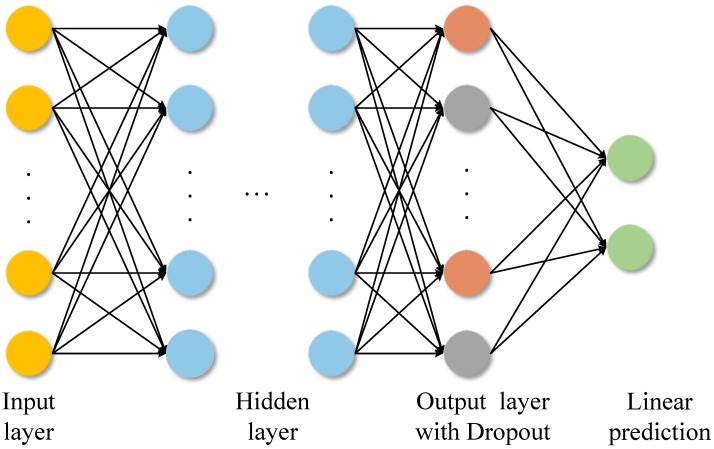
The MLP structure with dropout.

At each hidden layer *l*,as shown in Figure 5, we compute a hidden representation $H^{\left(l\right)}$ of dimension *H*, where *H* denotes the number of neurons in the hidden layer. The computation proceeds by first applying a linear transformation to the input, followed by layer normalization and a non-linear activation function.

This process is repeated for each subsequent hidden layer, and the forward propagation at layer *l* is given as follows:

(16) $$H^{\left(l\right)}=\sigma \left(\mathrm{LayerNorm}\left(W^{\left(l\right)}H^{\left(l-1\right)}+b^{\left(l\right)}\right)\right),$$

where $W^{\left(l\right)}$ and $b^{\left(l\right)}$ are the weight matrix and bias vector for layer *l*, respectively, and LayerNorm(·) denotes the layer normalization operation—its role is to normalize the input of each layer in the neural network, thereby improving training stability, accelerating convergence, and enhancing model performance. σ(·) represents a non-linear activation function Gaussian Error Linear Unit(GELU) [39]. The GELU activation smoothly scales each input according to the probability that it lies on the positive half-axis, thus circumventing the gradient discontinuity exhibited by ReLU in the neighborhood of zero. Moreover, because its output remains non-zero in the negative region, GELU mitigates the “dead-neuron” problem and retains representational capacity. Since the output varies continuously with the input magnitude, is fully differentiable, and adapts its amplitude, it provides the optimizer with finer-grained gradient information, which in practice often translates into faster and more stable convergence.

(17) Gelu(x)=xΦ(x)

(18) $$\Phi \left(x\right)\;=\;\frac{1}{2}\;\left(1+\mathrm{erf}\;\left(\frac{x}{\sqrt{2}}\right)\right)$$

(19) $$\mathrm{erf}\left(x\right)\;=\;\frac{2}{\sqrt{\pi }}\int_{0}^{x}e^{-t^{2}}\;dt$$

The output layer produces the estimated DOA values. If we assume *K* signal sources, the output layer will have *K* neurons, and its linear output can be expressed as follows:

(20) $$\hat{\theta }=H^{\left(L-1\right)}W_{L}+b_{L}$$

where $\hat{\theta }={\left[{\hat{\theta }}_{1},{\hat{\theta }}_{2},\ldots ,{\hat{\theta }}_{K}\right]}^{T}$ are the predicted DOAs. Here, *K* is the number of sources (which can be predetermined or estimated; in our case, we consider a fixed maximum number of sources for the regression output dimension).

To prevent overfitting during training, we incorporate a dropout mechanism in the MLP. In our implementation, we apply dropout with probability p=0.5 to the outputs of the final hidden layer; this means that during training, 50% of the neurons in that layer are randomly set to zero output. Regularization of dropout forces the network not to rely too heavily on specific neurons, thus improving the generalizability of the model. In practice, we found that using dropout in the MLP significantly reduced overfitting. After training, the dropout is turned off, and the full network is used for predicting DOA values from new data.

## 3. Results

### 3.1. Experimental Setup

We evaluated the performance of the proposed C-GNN-based DOA estimation method through simulations under a variety of challenging conditions and compared it with several baseline algorithms. To generate training and testing data, we constructed a simulated dataset of array snapshots. Each data sample corresponds to a set of snapshots of signals received by the sparse array, from which a covariance matrix input is computed. The source DOAs in each sample were drawn from a uniform distribution between −60° and 60°. The number of source signals was set to 2. The power of the source signal and the noise level were configured to achieve a range of SNRs from −5 dB to 10 dB.

All models are trained on a single GPU RTX 4060 using PyTorch for 100 epochs with a batch size of 512. To balance estimation accuracy and computational cost, the number of GNN layers was set to 8, and the number of MLP layers was designated as 6. The optimizer is AdamW with an initial learning rate of $5\times 10^{-4}$ and a weight decay of $1\times 10^{-4}$. A ReduceLROnPlateau scheduler halves the learning rate whenever the validation loss stops decreasing; we set patience = 2, threshold = $1\times 10^{-4}$.

We focused on low-snapshot scenarios: in most cases, the number of snapshots used to form each covariance matrix was limited, as few as 32 snapshots, up to at most 256 in some tests. A total of 300,000 samples were generated for training under these conditions, ensuring that the model was exposed to a wide variety of angles and noise levels. For performance evaluation, we generated additional test sets independent of the training data, using similar distributions of angles and SNRs. Each experiment was repeated with 100 Monte Carlo runs to obtain reliable performance metrics.

### 3.2. Effect of Snapshot Count

We first examine how the number of snapshots affects the accuracy of the DOA estimation of the proposed model at a fixed low SNR. In this test, we set the signal-to-noise ratio to −5 dB, used two signal sources, and varied the number of snapshots employed to compute the covariance matrix. The proposed C-GNN model was then used to estimate DOAs for each case.

**Figure 6:**
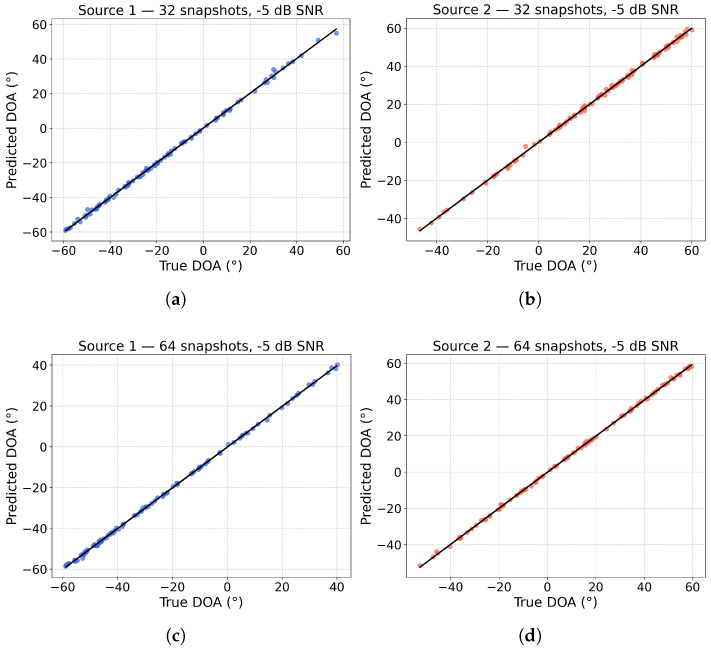

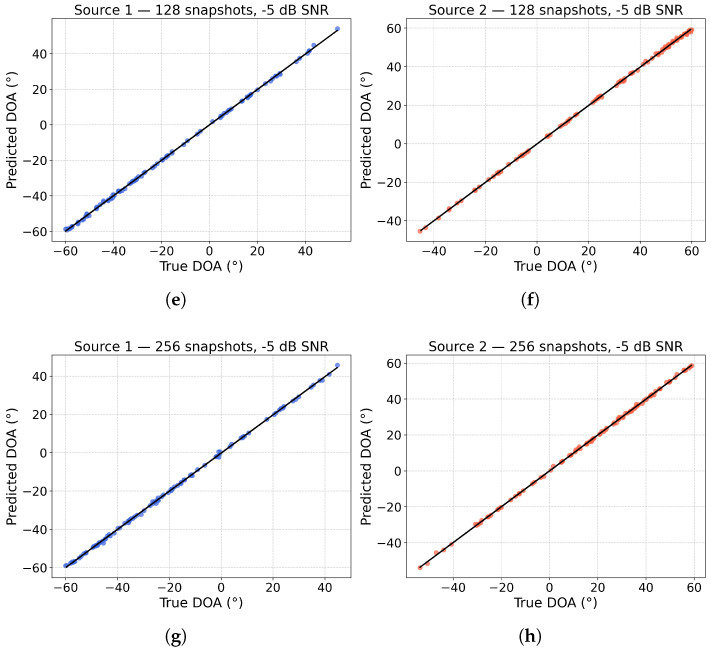
Scatter plots of DOA predictions at an SNR of −5 dB for various snapshot counts. Subfigures (**a**,**b**) correspond to 32 snapshots, (**c**,**d**) to 64 snapshots, (**e**,**f**) to 128 snapshots, and (**g**,**h**) to 256 snapshots.The x-axis shows the true angles, and the y-axis shows the predicted angles. In subfigures (**a**,**c**,**e**,**g**), blue dots represent the predicted DOAs of Source 1, while in subfigures (**b**,**d**,**f**,**h**), red dots indicate the predicted DOAs of Source 2.

Figure 6 shows scatter plots comparing the estimated DOAs to the true DOAs for snapshot counts ranging from 32 to 256, based on 100 Monte Carlo experiments. As the number of snapshots increases, the estimation accuracy improves significantly. Even with just 32 snapshots, the majority of the estimated angles closely align with the true values, demonstrating that the C-GNN can deliver accurate DOA estimates under limited data. With 64 and 128 snapshots, the estimates become more tightly clustered along the diagonal, indicating more accurate predictions. By 256 snapshots, the model’s estimates are highly precise, with minimal deviation from the true DOAs. These results underline the robustness of our approach: it delivers reliable DOA estimates even with a small number of snapshots, and its performance approaches near-optimal accuracy as the snapshot count increases.

### 3.3. Effect of SNR Under Low Number of Snapshots on the Performance of DOA Estimation

We compare the proposed C-GNN approach with several representative DOA estimation algorithms from the literature, including ESPRIT, Capon, a sparsity-based method using orthogonal matching pursuit (OMP), off-grid sparse Bayesian learning (OGSBL), long short-term memory convolutional neural network (LSTM-CNN) [40], and the GNN algorithm. For this comparison, we fixed the snapshot count to 32 and evaluated each method’s performance at SNR levels of −5, 0, 5, and 10 dB. The RMSE of the DOA estimates was computed for each algorithm at each SNR.

**Figure 7:**
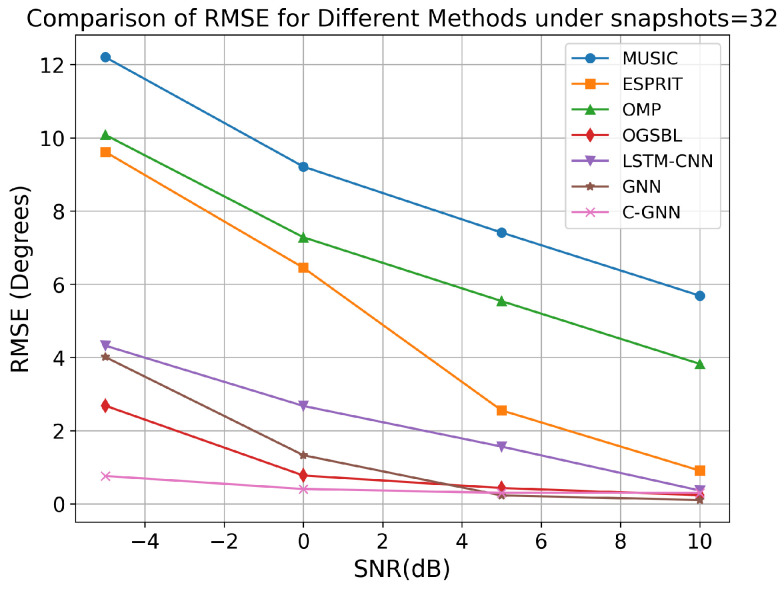
RMSE of different algorithms for 32 snapshots.

As shown in Figure 7, all of the algorithms show lower RMSE as SNR increases, but the improvement is most pronounced for the learning-based methods. In particular, at a SNR of −5 dB, OGSBL, LSTM-CNN, GNN, and the proposed C-GNN method achieve RMSEs much lower than traditional algorithms (MUSIC, ESPRIT, and OMP).

**Figure 8:**
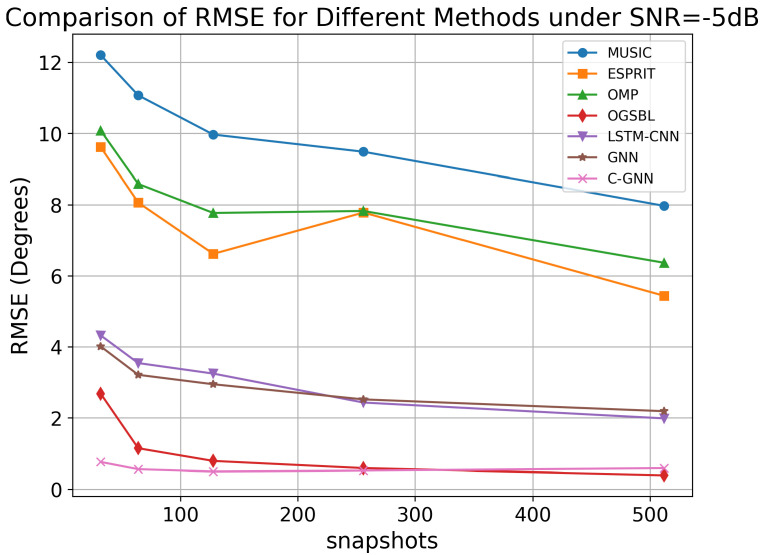
RMSE of different algorithms under SNR = −5 dB.

To evaluate the prediction performance of C-GNN under low SNR, the following experiment keeps the SNR fixed at −5 dB. We compare and analyze the RMSE values of various DOA algorithms for the range of 32 to 512 snapshots. The experimental results are shown in Figure 8.

As the number of snapshots increases from 32 to 512, the RMSE of most methods decreases, reflecting improved estimation accuracy. MUSIC and ESPRIT exhibit relatively higher RMSE values, particularly at lower snapshot counts, and they show a moderate improvement with increased snapshots. OGSBL, LSTM-CNN, and GNN also demonstrate improvements with increased snapshots, but their RMSE remains higher than that of C-GNN.

These results underscore the robustness of the proposed method: C-GNN can successfully extract and learn the signal features needed for accurate DOA estimation where conventional methods struggle, especially in the low-SNR, few-snapshot regime. This makes it a promising approach for scenarios in which only a limited number of snapshots can be collected or when real-time, on-the-fly DOA estimation is required.

In addition to RMSE, we evaluated the accuracy of the estimates in terms of a threshold criterion. Specifically, we defined an estimate to be accurate if the predicted DOA fell within ±0.5° of the true angle. We then measured the percentage of test trials that met this criterion for each algorithm under low-snapshot conditions.

**Figure 9:**
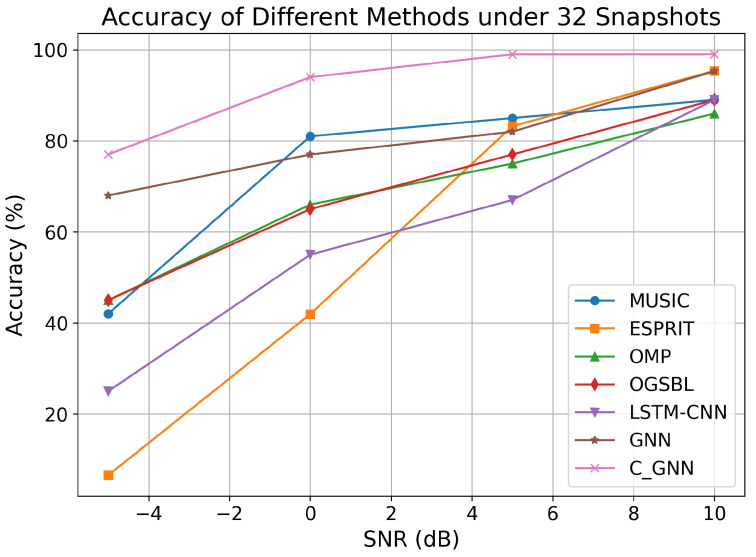
Accuracy of different algorithms under 32 snapshots.

From Figure 9, it can be observed that as the SNR increases for 32 snapshots, the prediction accuracy of each algorithm improves. However, in general, traditional algorithms exhibit lower accuracy and poor suppression ability in the low SNR range. The proposed C-GNN model achieved a higher accuracy than all the competing methods across the SNR range. For instance, at 32 snapshots and SNR = −5 dB, C-GNN produced a larger fraction of “within 0.5°” correct estimates, whereas traditional methods like MUSIC and ESPRIT had much lower accuracy under this stringent criterion.

On the other hand, learning-based algorithms are capable of learning more nonlinear representations, resulting in stronger robustness for LSTM-CNN and GNN. As shown in Table 1, the proposed C-GNN achieves nearly 80% accuracy under the condition of 32 snapshots and −5 dB, with the accuracy approaching 100% for SNR values above 5 dB. This demonstrates that C-GNN, based on the aggregation update principle, effectively learns the information collected between array elements, addressing the DOA estimation problem for sparse arrays with small snapshots and low SNR, while performing even better under high SNR conditions.

To provide a more intuitive picture of C-GNN’s prediction accuracy under low-snapshot conditions, we divide the angular range from –60° to 60° into 10° bins and plot a bar chart showing the model’s accuracy in each bin. This reveals at which angles the network is more prone to error and helps diagnose the sources of those errors.

**Figure 10:**
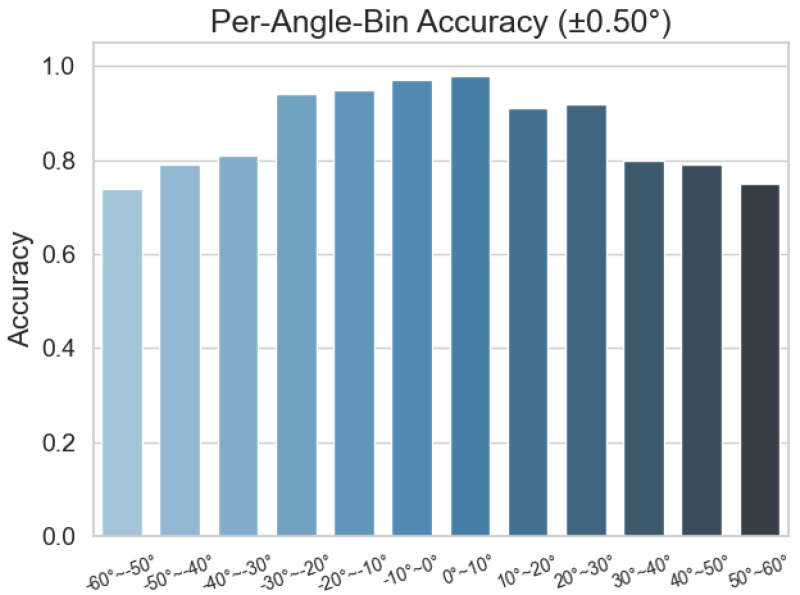
Angle interval prediction accuracy histogram.

Figure 10 shows that, with 32 snapshots and 0 dB SNR, the accuracy curve of C-GNN resembles a Gaussian distribution: predictions are roughly 95% accurate near 0°, but they fall to about 75% in the end-fire region. This difference can be traced to several intertwined factors. As the look angle approaches the end-fire direction, the array’s directional sensitivity gradually vanishes, which amplifies the inherent estimation error; meanwhile, the beam-pattern gain diminishes, lowering the effective SNR. End-fire angles are also under-represented in the training set, so the network encounters too few examples to learn their features well. These physical and data-driven causes together explain why the model performs noticeably worse at large absolute angles. In response to the above issues, we will continue to study this issue and improve it in its future work.

### 3.4. Actual Data Experiments

**Figure 11:**
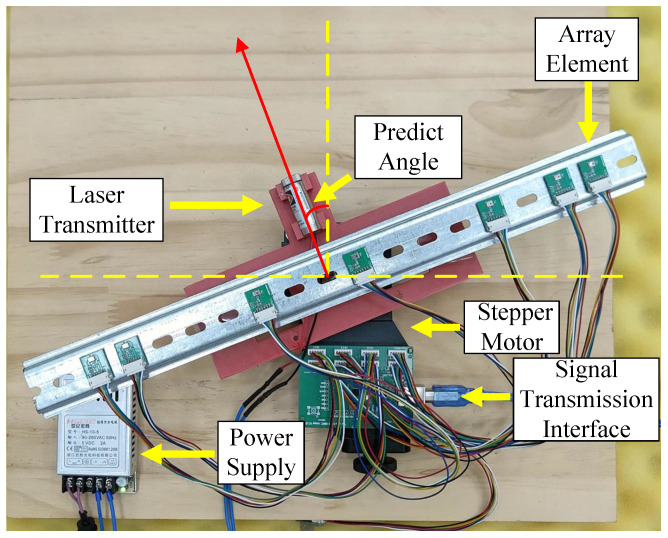
Signal receiving device.

To better validate the prediction performance of the model proposed in this paper, this section will use actual sound source signals for testing. Figure 11 shows the sound source localization device we designed. To ensure that the element spacing is within an acceptable margin of error, we installed tracks with fixed spacing holes on the device and arranged the elements according to the method described in Equation (8).

A laser emitter and a stepper motor are used to position the sound source. The signal data received by each element is transmitted to the computer through the signal transmission interface. To validate the effectiveness of our method, we subsequently process the signal waveforms received by each element to match the input form required by the algorithm proposed in this paper.

**Figure 12:**
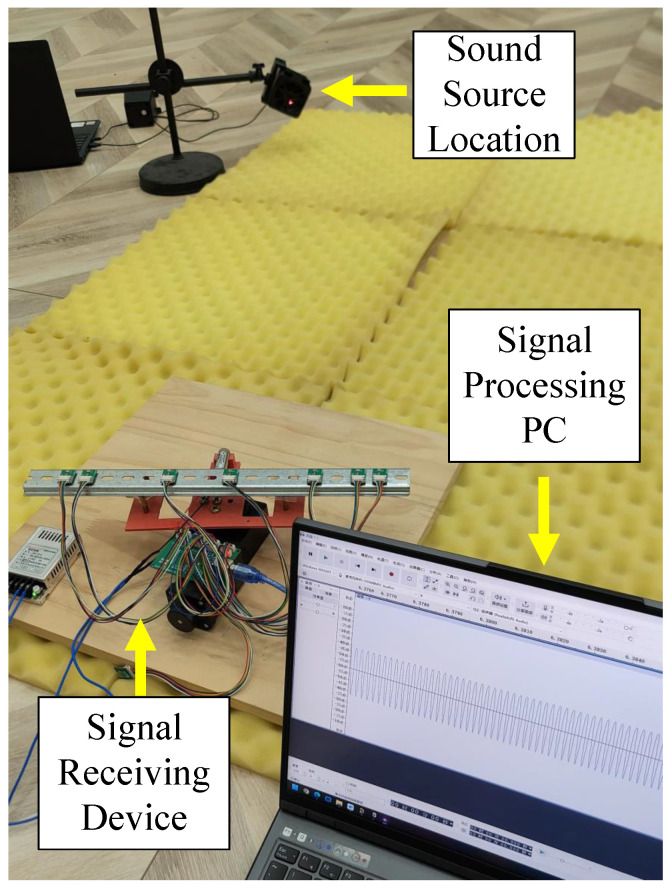
Schematic diagram of experimental setup.

Figure 12 shows the experimental setup. Due to space limitations, the experiments were conducted in a laboratory, and no measures were taken to reduce or prevent multipath effects. To reduce sound wave reflections and environmental noise interference, we laid acoustic foam in the open area.

After local processing, the estimated angle is fed back to the stepper motor control system, causing the laser to be directed to the corresponding angle, which is the predicted location of the sound source. In this experiment, the element spacing is set to 17 mm. To satisfy the Nyquist criterion, the input signal frequency is chosen to be 5000 Hz. Based on the determination of the signal’s far-field criterion, the distance between the sound source and the array is set to 1.5 m.

We recorded 32 snapshots of signal data from the initial time point, with the angle between the sound source and the signal receiver set to −60°, −30°, 0°, 30°, and 60°. Under the same input signal and angle conditions, we performed five independent repeated experiments and averaged the predicted angles. The experimental results are shown in Table 2:

It should be noted that due to the limitations in research cost and time, the element spacing may have some inherent errors in the design. Additionally, we used an engineering-level spirit level to determine the actual angle of the sound source, which may introduce some measurement errors. In the future, our research group will continue to optimize and supplement the physical testing components.

## 4. Conclusions

In summary, this work introduced a novel C-GNN architecture that integrates convolutional and GNN components to address DOA estimation for sparse arrays under limited snapshot conditions. The proposed method’s key contribution is the combination of 1D convolutions and graph convolutional layers, which together capture both local spatial patterns and global array geometry. By exploiting the difference coarray to form an VULA, the approach effectively emulates an expanded aperture with significantly increased degrees of freedom. Unlike conventional CNN or fully connected models that struggle with irregular array layouts, the graph-based component enables information aggregation across non-uniform sensor positions. Using real-valued covariance matrices as input and a regression-based MLP output, the C-GNN directly learns to map spatial correlation features to angle estimates without requiring large sample support. This hybrid methodology thus provides a robust solution for accurate DOA estimation in scenarios where there are low snapshots or low SNR, where traditional techniques often falter.

Looking forward, several extensions of this work are possible. First, we have demonstrated that C-GNN is well-suited for sparse arrays, and this adaptability may extend to a wider variety of antenna configurations. Additionally, due to its strong capability in handling small snapshot signals, the framework can be expanded to dynamic signal environments where the location of the signal source rapidly changes over time. Furthermore, exploring the feasibility of applying this algorithm in unsupervised or self-supervised learning is promising. An unsupervised training strategy would allow the model to learn directly from unlabeled array data, reducing reliance on simulated ground truth and potentially improving the model’s generalization ability. Finally, in the future, we plan to further optimize the existing physical experimental setup to explore the feasibility of our algorithm in real-world applications with more precise methods. By pursuing these directions, future research will further enhance the versatility and practicality of the proposed C-GNN approach.

## Figures and Tables

**Table 1:** DOA estimation accuracy at various SNR levels.

Method	−5 dB	0 dB	5 dB	10 dB
OGSBL	45.3%	65.2%	77.3%	89.1%
GNN	68.2%	77.0%	82.2%	95.3%
LSTM-CNN	25.3%	55.1%	67.8%	89.6%
C-GNN	77.6%	94.5%	99.7%	99.2%

**Table 2:** Actual and predicted angles with errors.

Experiment Number	Actual Angle (°)	Predicted Angle (°)	Error (°)
1	−60	−59.51	0.49
2	−30	−30.35	0.35
3	0	0.04	0.04
4	30	29.77	0.23
5	60	59.64	0.36

## Data Availability

The data presented in this study are available upon request from the corresponding author. The training dataset used in this article was obtained through simulation and is not a public dataset.
